# Cerebral blood flow changes in maintenance hemodialysis patients with restless legs syndrome and their clinical significance:a cross-sectional case-control study

**DOI:** 10.1186/s12883-024-03636-w

**Published:** 2024-04-16

**Authors:** Chen Li, Wei Sun, Linfang Xu, Cheng Chen, Li Fang, Yushang Tang, Qiaoyang Zhang, Haifeng Shi, Tongqiang Liu

**Affiliations:** 1 Department of Nephrology, The Affiliated Changzhou NO.2, People’s Hospital of Nanjing Medical University , Changzhou, Jiangsu China; 2 Graduate College, Dalian Medical University, Dalian, China; 3 Department of Radiology, The Affiliated Changzhou NO.2, People’s Hospital of Nanjing Medical University , Changzhou, Jiangsu China; 4 Hemodialysis Center, The Affiliated Changzhou NO.2, People’s Hospital of Nanjing Medical University , Changzhou, Jiangsu China; 5 Department of Psychology, The Affiliated Changzhou No. 2, People’s Hospital of Nanjing Medical University , Changzhou, Jiangsu China

**Keywords:** Maintenance hemodialysis, Restless legs syndrome, Arterial spin labeling, Cerebral blood flow

## Abstract

**Objective:**

Restless legs syndrome (RLS) stands as a prevalent neurological complication within maintenance hemodialysis (MHD) patients. However, the alterations in cerebral blood flow (CBF) among MHD-RLS patients remain uncharted. Through the utilization of the arterial spin labeling (ASL) technique, we evaluated the fluctuations in CBF within distinct brain regions and analyzed the risk factors for the development of RLS in MHD patients in the context of the clinic.

**Methods:**

Thirty-one MHD patients with concomitant RLS (MHD-RLS group) and thirty-one non-RLS patients matched based on age, gender, as well as cognitive function (MHD-nRLS group) were included. Through image preprocessing and data analysis, the changes in CBF values in distinct brain regions were obtained, and the CBF values of brain regions with substantial differences between the two groups were correlated with the RLS scores. Furthermore, the differences in baseline data were compared, and through the utilization of multifactorial logistic regression, the independent risk factors for the development of RLS were examined.

**Results:**

Compared with the MHD-nRLS group, the MHD-RLS group had increased CBF in the right superior temporal gyrus, reduced CBF in the right hippocampus, left middle frontal gyrus, inferior frontal gyrus of right triangle, middle frontal gyrus of left orbit, left precentral gyrus, and left precuneus. Only left precentral gyrus CBF were negatively correlated with RLS scores after correction for dialysis duration(*r* = -0.436, *P* = 0.016). Accordingly, multifactorial regression analysis by stepwise method yielded that the left precentral gyrus CBF values(OR: 0.968, 95%CI: 0.944–0.993, *P* = 0.012) remained an independent risk factor for RLS in MHD patients. In addition, the results showed that hemodialysis duration (OR: 1.055, 95%CI: 1.014–1.098, *P* = 0.008) and serum iron levels (OR: 0.685, 95%CI: 0.551–0.852, *P* = 0.001) were also risk factors for the development of RLS.

**Conclusion:**

Patients afflicted with MHD-RLS exhibit alterations in CBF across several brain regions. Notably, the left precentral gyrus might serve as a pivotal region influencing the onset of RLS among MHD patients. Furthermore, extended hemodialysis duration and a relative insufficiency in serum iron levels independently contribute as risk factors for RLS development within the MHD patient population.

## Introduction

Restless legs syndrome (RLS) constitutes a prevalent clinical neurological motor-sensory ailment. Its primary clinical attributes encompass an intense urge to shift the limbs prompted by discomfort in the legs, exacerbation or emergence of symptoms following periods of rest, partial or complete alleviation of symptoms through sustained activity, and an escalation or onset of symptoms during nighttime. This syndrome serves as a common neurological complication in individuals undergoing maintenance hemodialysis (MHD) [1]. Moreover, the prevalence of MHD patients with RLS (MHD-RLS) ranges from 19.4 to 57.3% [2], which is typically accompanied by symptoms including sleep disorders, cognitive impairments, cardiovascular events, anxiety, and depression, which deteriorates the patient’s quality of life and even worsens their prognosis [3]. Therefore, it is essential to identify clinical risk factors for the development of RLS in patients with MHD.

Recently, studies have indicated that arterial spin labeling (ASL) serves as a noninvasive, magnetic resonance technique for quantitatively assessing cerebral blood flow (CBF) perfusion status [4], in which CBF is a valid neuroimaging marker for assessing microvascular distribution, tissue metabolism, as well as organ function [5]. Furthermore, ASL is considered to have superior clinical applications due to the fact that it is non-invasive, safe, cost-effective, and reproducible. It has been indicated in the literature that ASL imaging technology has been able to visualize the CBF perfusion patterns of diseases including Alzheimer's disease and Parkinson's disease [6, 7] and examine the changes of CBF in local brain regions, which can assist in the diagnosis of the above-mentioned diseases by providing a visual foundation for the objective elucidation of pathophysiological disease mechanisms from an imaging perspective. Nonetheless, to date, there are fewer studies related to changes in CBF in patients with MHD-RLS. Consequently, research in this area is needed.

The objective of this study was to utilize the ASL technique to evaluate alterations in CBF across different brain regions in patients with MHD-RLS, to investigate the independent risk factors for the development of RLS in MHD patients, and to analyze their clinical significance.

## Objectives and methods

### Research subjects

This is a cross-sectional case-control study, and it included 280 patients undergoing regular maintenance hemodialysis at Changzhou Second People's Hospital of Nanjing Medical University from March 2022 to March 2023 were gathered and assessed. Inclusion Criteria were (1) age $\ge$ 18 years, (2) all MHD patients were diagnosed on the basis of the measurement of glomerular filtration rate(GFR), the GFR was less than 15 mL/min/1.73m^2^, (3) the hemodialysis duration in the patients was longer than 3 months, as well as a single hemodialysis duration session was approximately 4 h, (4)right-handedness, (5) complete clinical information, (6) no contraindications to MRI, (7) ability to complete relevant scale assessments. Exclusion criteria were (1) the presence of neurological or psychiatric disorders, including brain tumor, traumatic brain injury, stroke, epilepsy, schizophrenia, Parkinson and Alzheimer, (2) family history of RLS, (3) pharmacologic treatment of RLS, (4) history of neurologic drugs and alcohol dependence affecting RLS, (5) presence of anxiety and depression, (6)head movement artifacts interfering with experimental data acquisition or measurement. Based on inclusion and exclusion criteria, 31 patients with RLS (MHD-RLS group) and 31 MHD patients without RLS matched for age, sex, and cognitive function were included (MHD-nRLS group). The study was approved by the Ethics Committee of the Second People’s Hospital of Nanjing Medical University ([2022]KY005-01). Prior to the study, informed consent was obtained from the patients.

### Methods

#### Test flow chart

**Fig. 1:**
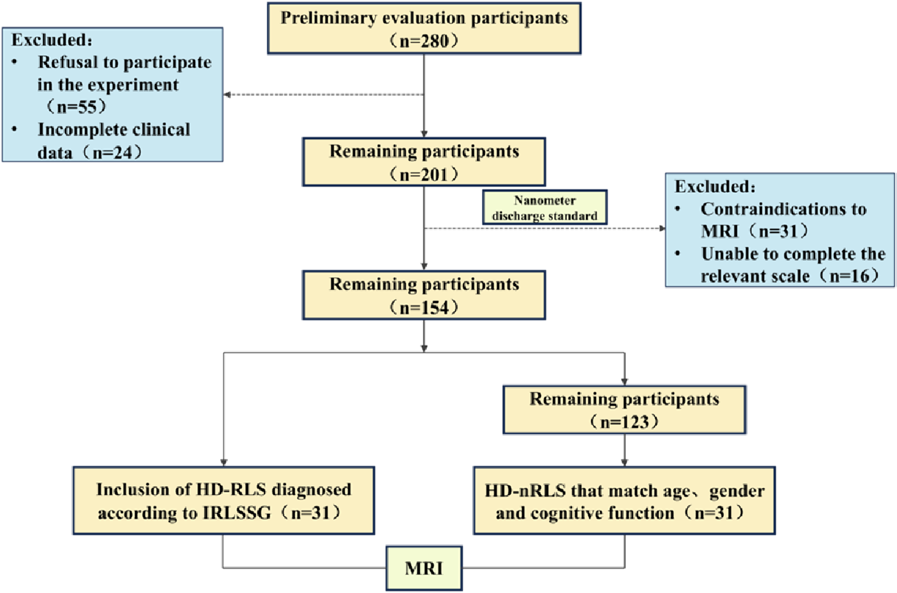
Flow diagram of the selection process of patients

Specific results are shown in Fig. 1.

#### General clinical information and laboratory tests

Collection of general clinical information on subjects, including gender, age, years of education, duration of hemodialysis, history of hypertension, history of diabetes, use of antihypertensive drugs or not etc. Furthermore, laboratory tests were performed on every subject within twenty-four hours prior to MRI examination, including systolic blood pressure(SBP), diastolic blood pressure(DBP), body weight gain, creatinine (Cr), blood urea nitrogen (BUN), uric acid (UA), white blood cell (WBC), red blood cell (RBC), hemoglobin (Hb), hematocrit (Hct), serum potassium (K^+^), serum sodium (Na^2+^), serum calcium (Ca^2+^), serum phosphorus (P), serum bicarbonate (HCO_3_^−^), total cholesterol (TC), triglyceride (TG), total protein (TP), albumin (Alb), alkaline phosphatase (ALP), high-density lipoprotein cholesterol (HDL-C), low-density lipoprotein cholesterol (LDL-C), parathyroid hormone (PTH), serum iron, serum ferritin, total iron binding force (TIBC), transferrin saturation (TS), folate, vitamin B12 (VitB12), urea clearance index (Kt/V) as well as C-reactive protein (CRP), etc.

#### Diagnosis of RLS

Diagnosis of RLS relies on the criteria outlined by the International Restless Legs Syndrome Study Group (IRLSSG) [8], necessitating the fulfillment of the following four symptoms. (1) an urge to move the legs, typically accompanied or caused by an uncomfortable or unpleasant sensation in the legs; (2) onset or intensification of this unpleasant sensation during rest or inactivity such as lying or sitting; (3) partial or total relief of the unpleasant sensation by movement, and (4) worsening or occurrence of the unpleasant sensation in the evening or night compared with daytime. Furthermore, RLS severity was assessed using the IRLSSG rating scale [9]. In our study, MHD-RLS patients were not treated with medications.

#### Neuropsychological assessment

All subjects completed the Montreal Cognitive Assessment (MOCA) scales 1 h after the MRI. Visuospatial, executive ability, memory, naming, attention, language, abstract thinking, and orientation are some of the cognitive dimensions measured by this scale, which is now widely used in clinical practice. Moreover, the total score is 30 points, when the subject has less than 12 years of education plus one point, over or equal to 26 points indicates no cognitive impairment, and less than 26 points is cognitive impairment.

#### Sleep quality assessment

The Pittsburgh Sleep Quality Index (PSQI) was used to assess the sleep quality of the subjects over the past month. Besides, the PSQI included 19 items that are composed of seven components: subjective sleep quality, sleep latency, total sleep duration, habitual sleep efficiency, sleep disturbance, use of sleep drugs, and daytime dysfunction. Each component is assigned a score between 0 and 3 points; the sum of the component scores is the total PSQI score; The total PSQI score ranges from 0 to 21; and higher scores indicate poorer sleep quality. Those with a total score greater than 5 are categorized as “poor sleepers”, whereas those with a score of 5 or less are categorized as “good sleepers”.

#### MRI acquisition

All subjects underwent scanning using a GE Discovery MR 750W 3.0 T scanner, and foam pads were added on both sides of the head to reduce head motion. Earplugs were employed to alleviate the noise from the MR scanner. During the scan, all subjects were required to lie quietly and flatly with their eyes closed yet awake and were asked to avoid falling asleep. Recent literature [10] has suggested that many neurological symptoms may be affected by dialysis treatment, therefore, all subjects were scanned 6–12 h before dialysis to eliminate its effects. To eliminate the effects of circadian rhythms, all subjects were scanned during the daytime and no RLS symptoms occurred during the scan. Additionally, MRI of the head was performed routinely on all subjects, the T2-FLAIR was utilized to exclude organic; high-resolution anatomic T1-weighted images were acquired with the three-dimensional brain volume imaging (3D-BRAVO) sequence [repetition time (TR) = 7.5 ms, echo time (TE) = 2.5 ms, inversion time (TI) = 450 ms, layer interval = 1 mm, flip angle (FA) = 15 ^∘^, the field of view (FOV) = 240 mm $\times$ 240 mm, slice thickness = 1 mm, number of scanned layers = 154.The whole scanning time was 3 min 51 s]. Followed by 3D-pcASL scanning, three-dimensional pseudo-continuous arterial spin labeling (3D-pcASL) sequence [repetition time (TR) = 5335 ms, echo time (TE) = 10.7 ms, post label delay (PLD) = 2525 ms, the field of view (FOV) = 240 mm $\times$ 240 mm, slice thickness = 4 mm, number of scanned slices = 36. The whole scanning time was 3 min 44 s].

#### MRI data analysis

Using the SPM12 toolkit based on the MATLAB 2018a platform, the following steps were taken to preprocess dates: (1) conversion of DICOM format to NFITI format; (2) each participant’s CBF images were co-registered to that person’s T1 anatomic maps, and the T1 maps have been normalized to Montreal Neurological Institute (MNI) space; (3) the CBF maps were subsequently brought to the MNI template by spatial transforms concatenation, with a voxel size for resampling of 3 $\times$ 3 $\times$ 3mm^3^; (4) finally, the normalized CBF maps were smoothed with a 5 $\times$ 5 $\times$ 5mm^3^ full width at half maximum (FWHM) Gaussian kernel, and the final run processed out is swCBF. The pre-processed CBF images were then analyzed with REST software to obtain CBF values.

### Statistical analysis

For baseline data, SPSS 26.0 was employed for statistical analysis. Furthermore, qualitative data were tested using $\chi 2$ test, expressed as frequency; utilizing t-tests on independent samples, quantitative data with a normal distribution were evaluated; expressed as ($\overline{X }\pm S$); non-normally distributed quantitative data were tested adopted the Mann–Whitney U-test, expressed as M(Q1, Q3); factors with *P* < 0.1 in the univariate analysis were included in the multivariate logistic regression by stepwise method for the purpose of analyzing the independent risk factors affecting the occurrence of RLS in patients with MHD. *P* < 0.05 was considered a statistically significant difference.

Differences in CBF values between the two groups were calculated by utilizing the DPABI toolbox. Gaussian random field (GRF) was corrected for multiple comparisons correction. Moreover, statistical thresholds were set at *P* < 0.05 for the voxel level and *P* < 0.05 for the clustering level (two-tailed). All coordinates are provided in the Montreal Neurological Institute (MNI) space. The CBF values of the brain regions displaying significant differences between the two groups were subject to correlation with the RLS scores, before and after correcting for dialysis duration. Additionally, logarithmic processing of the CBF values was undertaken to enhance the comparability of data correlations. Additionally, Pearson's correlation analysis follows a normal distribution, otherwise Spearman's correlation analysis. Correction for dialysis duration by partial correlation analysis.* P* < 0.05 was regarded as a statistically significant distinction.

## Results

### Results of CBF in brain regions between the two groups

**Fig. 2:**
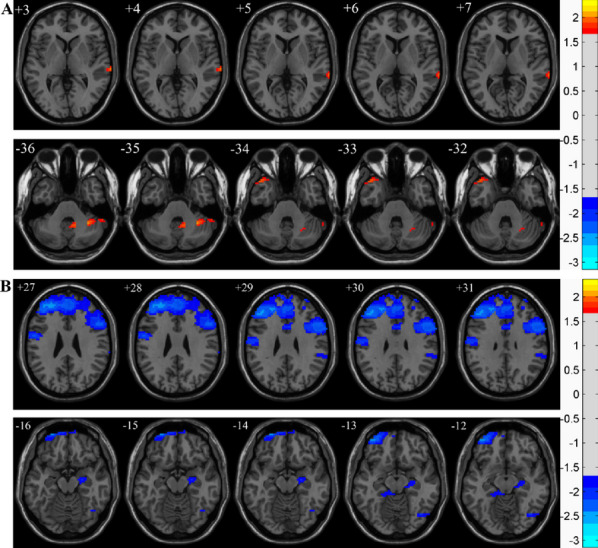
Brain regions exhibiting CBF differences between MHD-RLS and MHD-nRLS. **A **The red areas indicate significantly higher CBF values in the MHD-RLS group compared to the MHD-nRLS group. **B **The blue areas indicate significantly lower CBF values in the MHD-RLS group compared to the MHD-nRLS group

In comparison with the MHD-nRLS group, the MHD-RLS group had increased CBF values in the right superior temporal gyrus (*P* < 0.05), decreased CBF values in the right hippocampus, left middle frontal gyrus, inferior frontal gyrus of right triangle, middle frontal gyrus of left orbit, left precentral gyrus, and left precuneus (*P* < 0.05), and specific results are demonstrated in Table 1 and Fig. 2.

**Table 1:** Descriptions of statistically different brain regious between the two groups in CBF

Brain region	MNI			Cluster size	*Z* value	*P* value
	**X**	**Y**	**Z**			
Temporal Sup R	72	-24	3	10	2.034	< 0.05
Hippocampus R	24	-12	-15	23	-1.919	< 0.05
Frontal Mid L	-36	15	60	41	-2.453	< 0.05
Frontal Inf Tri R	39	36	9	92	-2.359	< 0.05
Frontal Mid Orb L	-24	63	-15	121	-2.558	< 0.05
Precentral L	-42	-9	57	251	-3.145	< 0.05
Precuneus L	-6	-36	36	108	-2.471	< 0.05

*MNI* Montreal Neurological Institute

### Correlation analysis

**Fig. 3:**
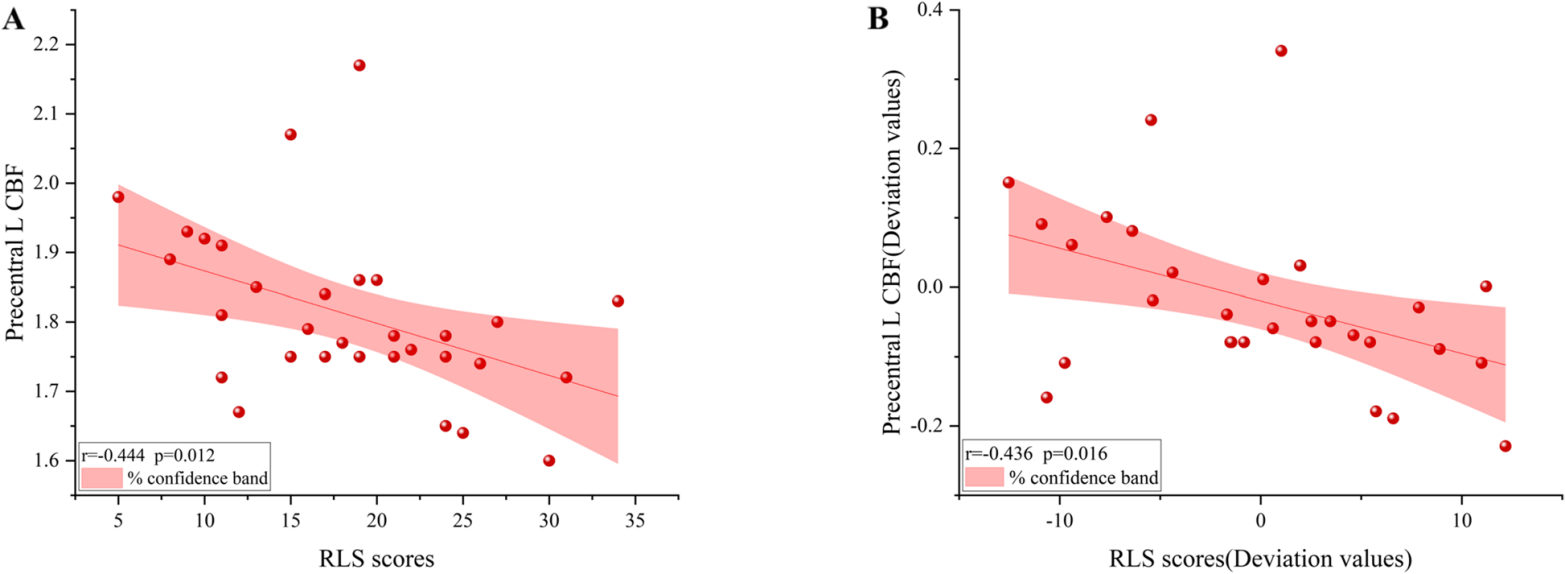
**A **Correlation analysis of the CBF values in left precentral gyrus and RLS scores. **B **Correlation analysis of the CBF values in left precentral gyrus and RLS scores after dialysis duration correction

Left precentral gyrus CBF values were negatively correlated with RLS scores in MHD-RLS patients (*r* = -0.444, *P* = 0.012), and this correlation remained significant after correction for dialysis duration(*r* = -0.436, *P* = 0.016). The remaining brain regions did not correlate significantly with RLS scores (*P* > 0.05). Specific results are shown in Fig. 3.

## Demographic, clinical, neuropsychological, and sleep quality results

No statistical differences were observed in age, gender, years of education, MoCA scores, hypertension, as well as diabetes between the participants in the two groups (*P* > 0.05). In the MHD-RLS group, the duration of hemodialysis exhibited a significantly greater length when compared to the MHD-nRLS group [26.0 (17.0,60.0) months *vs* 12.0 (6.0,24.0) months, *P* = 0.004]. Furthermore, the MHD-RLS group displayed notably reduced serum iron levels in contrast to the MHD-nRLS group (8.8 ± 3.0 umol/L *vs* 11.5 ± 3.6 umol/L, *P* = 0.002). TS was also markedly lower in the MHD-RLS group in comparison to the MHD-nRLS group (21.7 ± 7.8% *vs* 28.0 ± 10.7%, *P* = 0.010), whereas PSQI scores demonstrated a substantial elevation in the MHD-RLS group as opposed to the MHD-nRLS group (11.7 ± 3.3 scores *vs* 9.9 ± 3.3 scores, *P* = 0.038). Moreover, the difference in the remaining clinical indicators was not statistically significant (*P* > 0.05), and specific results are shown in Table 2.

**Table 2:** Demographic, clinical characteristics, MOCA and PSQI scores in the two groups

	**MHD-RLS** **(*n*=31)**	**MHD-nRLS** **(*n*=31)**	**t/Z/χ2**	***P* value**
Age(years)	50.7±9.4	46.3±11.6	-1.647	0.105^a^
Gender(male/female)	13/18	18/13	1.620	0.203^c^
Hypertension(n%)	24(77.4)	27(87.1)	1.005	0.316^c^
Diabetes(n%)	9(29.0)	6(19.4)	0.796	0.372^c^
Education(years)	9.0(6.0,12.0)	9.0(6.0,11.0)	-0.594	0.558^b^
Antihypertensive(n%)	22(71.0)	20(66.7)	0.132	0.717^c^
SBP(mmHg)	146.3±4.1	142.2±3.1	-0.807	0.423^a^
DBP(mmHg)	84.1±2.3	81.7±1.6	-0.861	0.393^a^
Body weight gain(%)	3.7±0.9	3.8±0.7	0.856	0.396^a^
MoCA(points)	25.0(21.0,26.0)	25.0(21.0,27.0)	-0.744	0.462^b^
HD duration(months)	26.0(17.0,60.0)	12.0(6.0,24.0)	-2.864	**0.004**^**b**^
BUN(mmol/L)	24.6(22.1,29.8)	23.7(20.2,28.5)	-0.873	0.387^b^
UA(umol/L)	426.1±70.4	417.1±67.1	-0.514	0.609^a^
Cr(umol/L)	996.6±215.3	984.9±207.0	-0.217	0.829^a^
K^+^(mmol/L)	4.6±0.8	4.4±0.9	-0.517	0.607^a^
Na^2+^(mmol/L)	139.5±3.6	140.4±3.5	0.960	0.341^a^
Ca^2+^(mmol/L)	2.2±0.2	2.2±0.3	-0.806	0.425^a^
HCO3^-^(mmol/L)	19.5±2.8	18.8±3.0	-1.018	0.313^a^
P(mmol/L)	2.0±0.6	1.9±0.6	-0.549	0.585^a^
WBC(10^9^/L)	6.1±2.0	6.7±1.9	0.038	0.230^a^
CRP(mg/L)	2.6(1.3,4.6)	2.3(1.7,5.6)	-0.169	0.870^b^
RBC(10^12^/L)	3.3±0.8	3.4±0.8	0.178	0.860^a^
Hb(g/L)	98.7±13.4	102.4±12.0	1.127	0.264^a^
Hct(%)	31.3±7.5	30.9±7.0	-0.181	0.857^a^
TP(g/L)	65.6±9.8	63.0±8.8	-1.105	0.274^a^
Alb(g/L)	39.0±6.6	37.6±6.0	-0.884	0.380^a^
TC(mmol/L)	4.1(3.3,4.6)	3.4(3.0,4.7)	-1.014	0.315^b^
TG(mmol/L)	1.7(1.1,2.4)	1.3(1.1,1.9)	-1.401	0.163^b^
HDL-C(mmol/L)	1.0(0.7,1.2)	0.9(0.8,1.0)	-0.366	0.719^b^
LDL-C(mmol/L)	2.0(1.7,2.6)	1.8(1.5,2.6)	-0.507	0.617^b^
ALP(U/L)	76.9(64.0,97.0)	79.0(65.0,103.0)	-0.606	0.550^b^
PTH (g/L)	240.8(183.6,806.0)	316.4(138.8,466.9)	-0.880	0.385^b^
Serum iron(umol/L)	8.8±3.0	11.5±3.6	3.166	**0.002**^**a**^
TIBC(umol/L)	42.3±10.7	44.2±12.4	0.653	0.516^a^
TS(%)	21.7±7.8	28.0±10.7	2.643	**0.010**^**a**^
Serum ferritin(ng/mL)	173.5±28.3	145.0±23.2	-0.779	0.439^a^
Folate(ng/mL)	5.9(4.2,9.1)	7.1(4.1,18.0)	-0.938	0.352^b^
VitB12(pg/mL)	672.1(460.6,889.9)	532.7(403.1,854.6)	-1.316	0.192^b^
PSQI(points)	11.7±3.3	9.9±3.3	-2.121	**0.038**^**a**^
Kt/V(ml-s-l/1.73m^2^)	1.3±0.1	1.4±0.1	3.652	0.276^a^

*SBP *Systolic blood pressure, *DBP *Diastolic blood pressure, *HD* *Duration* Hemodialysis duration, *MOCA *Montreal Cognitive Assessment, *PSQI *Pittsburgh Sleep Quality Index, *BUN *Blood urea nitrogen, *UA *Uric acid, *WBC *White blood cell, *RBC *Red blood cell, *TC *Total cholesterol, *TP *Total protein, *ALP *Alkaline phosphatase, *HDL-C *High-density lipoprotein cholesterol, *LDL-C *Low-density lipoprotein cholesterol, *PTH *Parathyroid hormone, *TIBC *Total iron binding force, *TS *Transferrin saturation, *Kt/V *Urea clearance index

^a^Independent Samples t-test

^b^Mann-Whitney U-test

^c^Chi-square test

### Logistic regression analysis

With combined RLS serving as the dependent variable, indicators with *P* < 0.05 in the baseline data were subjected to univariate analysis, and factors with *P* < 0. 1 in the univariate analysis and the left precentral gyrus CBF values were subjected to multifactorial logistic regression analysis, due to the fact that poor sleep and cognitive dysfunction were not causes of RLS, thus, it is not included in PQSI or MoCA. Statistical significance of left precentral gyrus CBF values, hemodialysis duration, serum iron levels, and TS was obtained by univariate analysis, and then stepwise multivariate analysis correction was applied, which showed that left precentral gyrus CBF values (OR: 0.968, 95%CI: 0.944–0.993, *P* = 0.012) were still an independent risk factor for the occurrence of RLS in MHD patients. Meanwhile, hemodialysis duration (OR: 1.055, 95%CI: 1.014–1.098, *P* = 0.008), serum iron levels (OR: 0.685, 95%CI: 0.551–0.852, *P* = 0.001) were also risk factors for the occurrence of RLS. Specific results are illustrated in Table 3.

**Table 3:** Multivariate linear regression analysis of indicators between two groups

Variables	Univariate analysis		Multivariate analysis	
	**OR(95%CI)**	***P* value**	**OR(95%CI)**	***P* value**
HD duration	1.035(1.007–1.065)	0.016	1.055(1.014–1.098)	**0.008**
Serum iron	0.786(0.664–0.929)	0.005	0.685(0.551–0.852)	**0.001**
TS	0.927(0.871–0.986)	0.017		
Precentral L CBF	0.974(0.956–0.994)	0.010	0.968(0.944–0.993)	**0.012**

Multivariate Logistic regression analysis using the stepwise method resulted in *P* = 0.205 for the Hosmer-Lemescho, indicating a good model fit goodness-of-fit effect

## Discussion

In this study, we determined that there were changes in CBF in several brain regions in patients with MHD-RLS, in which decreased CBF in the left precentral gyrus possible valid neuroimaging marker for MHD-RLS. Similarly, long dialysis duration and relative deficiency of serum iron levels are independent risk factors for MHD-RLS.

Neuroimaging techniques have been adopted to assess the pathophysiologic mechanisms of RLS, and ASL has been used in the present study to provide evidence of CBF changes associated with RLS symptoms in MHD patients. Accordingly, we determined that MHD-RLS patients have abnormal CBF changes in multiple brain regions. In our study, the MHD-RLS group had increased CBF values in the right superior temporal gyrus in comparison to the MHD-nRLS group, indicating that the increased blood flow may be due to abnormal conditioning to sensory stimulation in RLS patients, which may be associated with their ill feelings in the legs [11]. Besides, the temporal lobe is part of the default mode network (DMN), the research results of Lee et al. recommend that the resting-state connectivity of the brain in the thalamus, bilateral lingual gyrus, as well as right middle temporal gyrus is a noteworthy finding in RLS, which is unaffected by dopamine agonist treatment [12], which offers conceptual support for our findings. Additionally, diminished CBF values were identified in the right hippocampus, left middle frontal gyrus, inferior frontal gyrus of the right triangle, middle frontal gyrus of the left orbit, left precentral gyrus, and left precuneus. This decrease in CBF could potentially be linked to the impairment of cerebrovascular autoregulation mechanisms. Moreover, the brain is the body's highest oxygen-consuming organ, and it has its regulatory mechanisms. Under normal conditions, decreasing oxygen levels promote vasodilation, which increases CBF and oxygen delivery to the brain, nevertheless, through the results of Salminen et al., the manifestation of peripheral hypoxia in RLS patients was observed to be highly related to the severity of RLS, and the hypothesis of microvascular abnormality in RLS was also supported [13], Thus, RLS causes a persistent chronic hypoxic state, which contributes to cerebral vascular damage and the loss of self-regulatory mechanisms, as well as ultimately results in a decrease in CBF [14]. Moreover, the frontal, precuneus, and precentral gyrus belong to the sensory-motor network (SMN), and the hippocampus is part of the limbic system, patients with MHD-RLS have reduced CBF in the sensorimotor network, indicating sensory-motor symptoms with abnormal regulation and a strong desire to move both lower extremities as a result of abnormal limbic system regulation [15].

Our study further found, left precentral gyrus CBF values were negatively correlated with RLS scores, and there was no significant correlation in the remaining brain regions, suggesting that the left precentral gyrus CBF values were related to the severity of RLS. We hypothesized that abnormal CBF in this brain region may be involved in the underlying pathophysiological mechanisms in patients with MHD-RLS. Except for the precentral gyrus is the predominant element of the primary motor cortex and is associated with controlling motor performance in RLS [16]. Hypoperfusion of CBF in this region leaded to vascular damage, causing altered cerebral regeneration and reduced volume in the precentral gyrus, which ultimately produced a lack of control of leg movements in patients with MHD [17]. Similarly, Wang et al. demonstrated by voxel-based morphometry (VBM) analysis that the gray matter volume (GMV) of the left primary motor cortex was reduced in HD-RLS, primarily in the precentral gyrus, demonstrating that this abnormal decreased GMV in the sensorimotor cortex provides evidence for a sensory processing disorder in RLS [18]. The above findings may indicate that the symptoms of RLS are primarily related to the motor and somatosensory cortex, which, in connection with the present study, may validate further the hypothesis that RLS is linked to alterations in spontaneous brain activity and brain perfusion in the basal ganglia-thalamic-cortical circuit (CST) [11]. The CST, as a white matter motor pathway starting at the cortex that terminates on motor neurons in the spinal cord, is an essential structure that contains many communication pathways, controlling movements of the limbs and trunk [19]. There was also some literature on other imaging methods, such as, functional magnetic resonance (fMRI), Liu et al. found that patients with idiopathic RLS have reduced amplitude of low-frequency fluctuations(ALFF) in the sensorimotor and visual processing systems [20]; and with iron-sensitive MRI, Han et al. obtained, by quantitative weighted imaging (QSM) analysis, reduced magnetization of the caudate and shell nuclei in HD-RLS patients [21]. In the future, we can also increase research in these areas. In conclusion, cerebral perfusion imaging measurements could potentially serve as an early detection biomarker for MHD-RLS, aiding in the identification of alterations in sensory-motor pathways linked with potentially anomalous sensorimotor function, and facilitating the monitoring of treatment effectiveness within MHD-RLS populations.

The duration of dialysis in MHD patients was substantially associated with the occurrence of RLS, which is consistent with the results of a study by Tsai L H et al. [2], whose results demonstrated that individuals undergoing dialysis for more than 5 years exhibited a 2.32 times higher likelihood of developing RLS compared to those with less than 5 years of dialysis history. Furthermore, the intensity of RLS demonstrated a positive correlation with the duration of dialysis. However, some studies have also found no significant association between the onset of RLS and the duration of dialysis, and it is considered that this may be related to patient heterogeneity [22]. In our study, dialysis times was significantly longer in the MHD-RLS group than in the MHD-nRLS group, as well as independently constituting a risk factor for the development of RLS. Similar results were obtained by Wang et al. [21]. It has been revealed in the literature that the duration of dialysis in MHD patients is associated with increased oxidative stress as well as decreased serum antioxidant enzymes [23], oxidative stress is prevalent in chronic kidney disease and may heighten the risk of cardiovascular disease, cachexia, anemia, and other comorbid conditions, oxidative stress is an imbalance between pro-oxidant production and antioxidant defense mechanisms. Besides, the pro-oxidant and antioxidant activities of patients in distinct stages of chronic kidney disease are divergent, while hemodialysis patients have the most impaired renal function. Consequently, they have the highest amount of oxidative stress, which contributes to the elevation of serum markers F2-isoacids and a corresponding notable decline in antioxidant activity, predominantly in the decline of metabolic enzyme and glutathione peroxidase activity [24], and likewise due to the fact that RLS causes a persistent chronic hypoxic state, which may longer dialysis times may increase the incidence of RLS by promoting the development of oxidative stress. In addition, Salman SM et al. noted the presence of hyperexcitability of motor nerves in patients with MHD and the occurrence of peripheral neuropathy in patients with MHD in correlation with the duration of dialysis [25], which may also reflect the higher susceptibility to RLS as the duration of dialysis increases in patients with MHD.

We identified notably diminished serum iron levels within the MHD-RLS group in comparison to the MHD-nRLS group and that serum iron levels independently contributed as a risk factor for the onset of RLS. Previous studies have also reported that low serum iron levels may be a risk factor for RLS [26] and that there is a positive correlation between body iron stores, as measured by serum iron and ferritin levels, and symptom severity [27]. However, there is also a part of the literature that suggests that the majority of patients with RLS do not present with iron deficiency considerations may be related to the long-term use of iron therapy in patients with MHD [22, 28]. The literature suggests that the possible pathogenesis of the disease is iron deficiency with damage to dopaminergic neurons [29]. Furthermore, iron is a cofactor for tyrosine hydroxylase, an enzyme that regulates tyrosine metabolism as well as affects dopamine synthesis [30], and low levels of iron may induce or exacerbate RLS by decreasing the activity of the dopamine system, ultimately, the body's speed of movement and exercise endurance decreases, which in turn inhibits motor nerve conduction and increases the incidence of RLS [31]. Additionally, previous studies have indicated that decreased iron in the brain of RLS patients also causes downregulation of the dopaminergic system, particularly in the substantia nigra, the nucleus accumbens, as well as the medial thalamic area, which in turn leads to an increase in the excitability of the spinal cord, which ultimately manifests itself in RLS symptoms [32]. Secondly, whether correction of the lack of serum iron levels can enhance the symptoms of RLS was addressed by Park et al. Their results indicated that repeated intravenous iron sucrose is an effective treatment for secondary RLS associated with iron deficiency, which also suggested that iron deficiency may be associated with the development of RLS in patients with MHD [26].

Nonetheless, there are some limitations to our study. Firstly, its cross-sectional nature restricts the ability to establish definitive causal relationships. Secondly, the diagnosis of patients with MHD-RLS was based on questionnaires and clinical presentation without polysomnographic monitoring recordings, which may have overlooked cases of mild RLS among the study participants. Thirdly, according to the literature, hemoglobin and ferritin are mostly recognized as clinical risk factors for the development of RLS, which was not reflected in our results, which may be related to the insufficient sample size or the heterogeneity of individuals. Fourthly, the enrolled patients were on dialysis for a relatively short period of time, which may have introduced some bias. Finally, the relatively small sample size could introduce bias in the assessment of RLS prevalence and associated clinical risk factors among MHD patients. Therefore, an expanded sample size would be necessary for more comprehensive insights.

## Conclusion

Changes in CBF in the presence of multiple brain regions in MHD-RLS patients, the left precentral gyrus may play a significant role in the development of RLS in MHD patients, and the long duration of hemodialysis and relative deficiency of serum iron levels are independent risk factors for the development of RLS in MHD patients. This provides an important basis for exploring the mechanism of the occurrence of RLS in patients with MHD and clinical practice,and contribute to early recognition and treatment of MHD-RLS, thereby enhancing patients’ quality of life.

## Data Availability

No datasets were generated or analysed during the current study.

## Abbreviations

RLS: Restless legs syndrome
MHD: Maintenance hemodialysis
ASL: Arterial spin labeling
CBF: Cerebral blood flow
IRLSSG: International Restless Legs Syndrome Study Group
DMN: Default mode network
SMN: Sensory-motor network
VBM: Voxel-based morphometry
GMV: Gray matter volume
CST: Basal ganglia-thalamic-cortical circuit
ALFF: Amplitude of low-frequency fluctuations
QSM: Quantitative weighted imaging
